# Symmetry Lasts Longer Than Random, but Only for Brief Presentations

**DOI:** 10.1177/2041669516676824

**Published:** 2016-11-16

**Authors:** Ruth Ogden, Alexis D. J. Makin, Letizia Palumbo, Marco Bertamini

**Affiliations:** School of Natural Sciences and Psychology, Liverpool John Moores University, UK; Department of Psychological Sciences, University of Liverpool, UK; Department of Psychological Sciences, University of Liverpool, UK; Department of Psychology, Liverpool Hope University, UK; Department of Psychological Sciences, University of Liverpool, UK

**Keywords:** symmetry, time, subjective duration, internal clock, arousal

## Abstract

Previous research has shown that explicit emotional content or physical image properties (e.g., luminance, size, and numerosity) alter subjective duration. Palumbo recently demonstrated that the presence or absence of abstract reflectional symmetry also influenced subjective duration. Here, we explored this phenomenon further by varying the type of symmetry (reflection or rotation) and the objective duration of stimulus presentation (less or more than 1 second). Experiment 1 used a verbal estimation task in which participants estimated the presentation duration of reflection, rotation symmetry, or random square-field patterns. Longer estimates were given for reflectional symmetry images than rotation or random, but only when the image was presented for less than 1 second. There was no difference between rotation and random. These findings were confirmed by a second experiment using a paired-comparison task. This temporal distortion could be because reflection has positive valence or because it is processed efficiently be the visual system. The mechanism remains to be determined. We are relatively sure, however, that reflectional patterns can increase subjective duration in the absence of explicit semantic content, and in the absence of changes in the size, luminance, or numerosity in the images.

## Introduction

It is well established that subjective estimates of duration can differ from actual stimulus duration. For example, the semantic content of an image can alter its perceived duration, as can physical properties like motion, numerosity, luminance, and size. This has been studied extensively with emotional images (see Droit-Volet & Meck, 2007 for review), and it has been found that images associated with fear are judged as lasting for longer than neutral images presented for the same duration (Gil & Droit-Volet, 2012). For example, an image of an angry face is judged to have been present for longer than an image of a neutral face (Droit-Volet, Brunot, & Niedenthal, 2004). Positively valenced affective images can also distort perceived duration. Typically, high-arousal positive images are associated with relatively shorter duration estimates, whereas low-arousal images are associated with relatively longer duration estimates (Angrilli, Cherubini, Pavese, & Mantredini, 1997; Smith, McIver, Di Nella, & Crease, 2011). These valence-arousal effects can be understood within the framework of scalar expectancy theory (SET; Gibbon, Church, & Meck, 1984).

**Figure 1.**
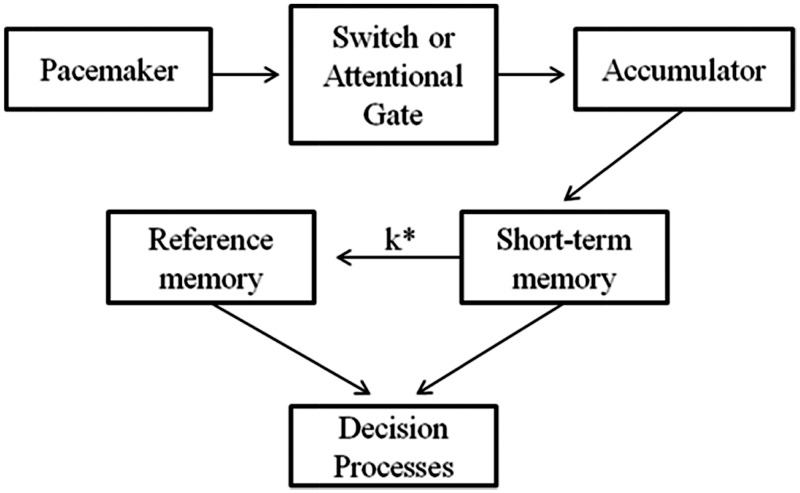
Schematic diagram of scalar expectancy theory (SET: Gibbon, Church, & Meck, 1984).

SET (Figure 1) proposes that humans use an internal pacemaker-accumulator clock to judge the duration of events. The pacemaker emits pulses at regular intervals. At the start of a to-be-timed event, the switch between the pacemaker and the accumulator closes, and ticks are transferred from the pacemaker to the accumulator. When the event ends, the switch opens and accumulation ceases. The number of ticks accumulated forms the subjective representation of duration, so that more ticks equals more time. The number of accumulated ticks can then be compared with other duration representations stored in memory to enable timed behaviour.

Like many cognitive models, SET is somewhat metaphorical, particularly as it is unclear how a pacemaker-accumulator clock would be implemented neurally. Furthermore, there is no consensus about whether there is a single pacemaker in the brain, or whether there are many such internal clocks (Johnston, Arnold, & Nishida, 2006; Van Rijn & Taatgen, 2008). Despite uncertainty about neural implementation, the SET framework has been very influential, and it can explain many findings from human psychophysics, animal timing, and pharmacological studies (reviewed in Buhusi & Meck, 2005 or Coull, Cheng, & Meck, 2011). Importantly, alterations of subjective duration can be divided into effects on pacemaker speed and effects on the switch between the pacemaker and accumulator.

When participants verbally estimate the duration of stimuli, subjective duration increases with actual duration. We can measure the slope and intercept of this relationship with linear regression. Changes in pacemaker speed typically alter the slope (e.g., Penton-Voak, Edwards, Percival, & Wearden, 1996; Wearden, Edwards, Fakhri, & Percival, 1998). That is, the difference between subjectively shorter and longer conditions is multiplicative and grows with actual stimulus duration. Pacemaker speed is thought to be arousal sensitive (although arousal is not always defined or recorded independently). For example, pacemaker speed can be increased by dopaminergic agonists such as amphetamine or decreased with antagonists like haloperidol (Meck, 1983). Fearful stimuli (Fayolle, Gil, & Droit-Volet, 2015; Gil & Droit-Volet, 2012; Ogden, Redfern, Moore, & McGlone, 2015) and low-arousal positive images therefore lengthen duration estimates because of the arousal they produce and the associated increase in pacemaker speed. Although pacemaker effects are typically multiplicative, recent evidence suggests that emotional arousal effects on timing may extinguish at longer durations (greater than 1 second; Gil & Droit-Volet, 2012), meaning that slope effects are not ubiquitous with pacemaker output change.

The operation of the switch is governed by attention (Zakay & Block, 1997). Anything that reduces attention to time stops or reduces the transfer of ticks from the pacemaker to the accumulator, and fewer ticks are ultimately accumulated, resulting in a shortening of subjective duration. This is typically thought to manifest as an intercept effect. Reduced attention to time can explain why high-arousal positively valenced images and high-arousal negative images produce opposite effects on time perception (Angrilli et al., 1997). The high-arousal positive valenced images used in Angrilli et al. (1997) depicted naked people and erotic scenes, and the appetitive nature of these images, although arousing, detracts attention from ongoing tasks (Most, Smith, Cooter, Levy, & Zald, 2007). In the case of timing, this distraction reduces the accumulation of ticks leading to a shortening of duration.

While attention and arousal, as defined by SET, are able to explain the various effects of emotion on time perception, it should be noted that these explanations are somewhat unfalsifiable. For example, high-arousal negative valence images from the international affective picture system are both arousing and attention grabbing and could therefore theoretically lead to both over and underestimations of duration. Indeed, the absence of objective measures of attention and arousal during emotion-timing studies means that explanations can be applied post hoc based on the semantic content of the stimulus.

Differing semantic content is not the only variable which complicates the understanding of distortions to time. It is also unclear to what extent the nonsemantic physical image properties are influencing perceived duration (e.g., complexity, size, and luminance). Image complexity, for example, as defined by algorithms that extract edges and symmetries, has been shown to influence duration estimates when explicit semantic content is present (Cardaci, Di Gesù, Petrou, & Tabacchi, 2006, 2009; Folta-Schoofs, Wolf, Treue, & Schoofs, 2014) but not when it is absent (Palumbo, Ogden, Makin, & Bertamini, 2014). Indeed, even in the absence of explicit semantic content, the number of discrete items in a stimulus (Xuan, Zhang, He, & Chen, 2007), stimulus luminance (Goldstone & Goldfarb, 1964), stimulus size (Xuan, et al., 2007; Thomas & Cantor, 1975), and stimulus colour (Gorn, Chattopadhyay, Sengupta, & Tripathi, 2004) have all been shown to influence perceived duration. Many of these purportedly affect timing because they increase arousal. Therefore, in studies in which images are used (e.g., the international affective picture system), but these factors are not explicitly controlled across conditions, it is unclear whether nonaffective physical properties of the images contribute to the effects observed. These concerns are somewhat allayed by the use of facial images expressing different emotional expressions as stimuli (Droit-Volet et al., 2004). However, the use of pink ovals as control stimuli (rather than neutral expressions), coupled with the distinct neural circuitry used in the processing of emotional faces (Kanwisher, McDermott, & Chun, 1997), means that further investigation is warranted to establish whether stimulus valence can influence perceived duration in the absence of these potential confounds.

Visual symmetry provides an opportunity to study distortions to subjective duration while controlling for semantic content, differences in complexity, numerosity, colour, size, and luminance. Abstract visual symmetry is rated positively by most participants (Eisenman, 1967; Eysenk, 1941; Jacobsen & Hofel, 2002; Makin, Pecchinenda, & Bertamini, 2012). It is known that reflectional symmetry is associated with positive valence words in implicit association tests (Bertamini, Makin, & Rampone, 2013). This is unlikely to be a cultural whim; many species have a preference for symmetrical mates (Møller & Thornhill, 1998) and symmetrical foods (Wignall, Heiling, Cheng, & Herberstein, 2006). It could be that phenotypic symmetry is a truthful indicator of health and genetic quality (Grammer, Fink, Møller, & Thornhill, 2003), and there is some evidence that humans are sexually attracted to symmetrical faces (Rhodes, Proffitt, Grady, & Sumich, 1998) and symmetrical bodies (Bertamini, Byrne, & Bennett, 2013). However, the “good genes” theory of symmetry preference has been questioned because the size of fluctuating asymmetries is often below perceptual discrimination thresholds (Swaddle, 1999), and there is not necessarily a reliable effect of symmetry on facial attractiveness once correcting for publication bias (van Dongen, 2011). Alternatively, it may be that symmetry is liked simply because symmetry is quickly and fluently processed (Reber, 2012), producing maximal visual responses (Enquist & Johnstone, 1997).

Palumbo, Ogden, Makin, and Bertamini (2015) recently demonstrated that symmetrical images are judged to have been presented for longer than random images of the same objective duration, even though the same patterns were evaluated positively by the participants. While this finding is consistent with the subjective lengthening reported for low-arousal positive stimuli with explicit semantic content (Angrilli et al., 1997; Droit-Volet et al., 2004), the finding was not the focus of Palumbo et al. (2015). Here, we followed up the preliminary findings of Palumbo et al. (2015) in two new experiments. In Experiment 1, we measured verbal duration estimates for regular and random patterns that were presented for 500, 750, 1000, 1250, or 1500 ms. In Experiment 2, we employed a different protocol, the paired-comparison task, in which participants indicate which of two stimuli (A and B) lasted for longer. This method does not require participants to apply numerical labels (e.g., 1000 ms) to the stimulus, thus confirming that the finding is not an artefact of verbal estimation itself.

**Figure 2.**
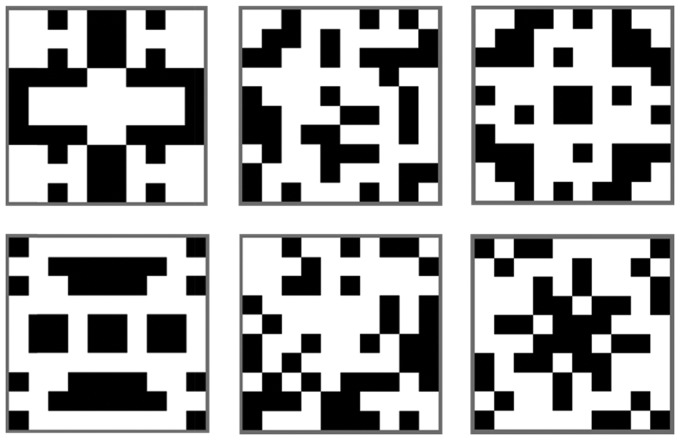
Illustration of the image types used in the experiments. Left panel: reflectional symmetry. Middle panel: random. Right panel: rotational symmetry. Every trial used a different example without any repetition.

In Experiment 1, we also explored two kinds of symmetry, reflection and rotation (Figure 2). Reflectional symmetry and rotational symmetry are equally regular in the mathematical sense, but reflection is more salient for human observers (Mach, 1886/1959; Palmer & Hemenway, 1978; Royer, 1981; van der Helm & Leeuwenberg, 1996). It could be that temporal distortions are specific to the more obvious reflectional symmetry. Alternatively, it could be that any kind of visual regularity increases subjective duration.

## Experiment 1

### Method

#### Participants

Twenty-two participants (M age = 22.16 years, *SD* = 1.36) took part in Experiment 1. All participants had normal or corrected-to-normal vision. Participants received £5 for participation. The experiment lasted 25 minutes. Participants provided written consent before taking part. Both experiments reported in this article were approved by the Ethics Committee of the Liverpool John Moores University and were conducted in accordance with the Declaration of Helsinki (2008).

#### Stimulus and apparatus

Example stimuli are shown in Figure 2. They were designed to be similar to those used in our previous work (Palumbo et al., 2015) and by Royer (1981). Novel patterns were generated afresh on every trial using the same algorithm, implemented in Python using open source *Psychopy* software (Peirce, 2007). Stimuli consisted of a matrix with 10 × 10 squares (320 × 320px, visual angle = 10.45° × 13.34°). Of the 100 squares, 40 were black (32 × 32 px) and the others white. The reflection patterns had two axis of symmetry: horizontal and vertical. The rotation patterns were 90° rotations. This design meant that the information in a single quadrant was identical in reflection, rotation, and random trials. Regularity was determined by the spatial relationship of elements across the quadrants. Importantly, the luminance, density, and size of these patterns were all equal, so changes in perceived duration can only be attributed to regularity as such, or to differences in other low-level visual properties such as luminance.

### Procedure

We employed a 3 × 5 within-subjects design with image type (reflectional symmetry, rotational symmetry, and random) and presentation duration (500, 750, 1000, 1250, and 1500 ms) as independent variables. The dependent variable was the estimated duration. There were 12 repeats of each condition, giving 180 experimental trials in total.

There were an additional 30 filler trials (10 reflections, 10 rotations, and 10 random) where the duration was selected at random from a uniform distribution ranging from 250 to 1750 ms. The filler trials prevent participants from overlearning the five durations in the experimental design. All 210 trials were presented in a random order. The experiment was divided into 10 blocks of 21 trials, so participants could have take breaks between blocks.

Participants were seated approximately 60 cm from the computer screen. They were instructed that they would be presented with images, and that their task was to estimate, in milliseconds, how long each image was displayed. Participants were informed that their estimates should be between 250 and 1750 ms. At the start of a trial, a fixation cross was presented in the centre of a grey background for 1000 ms, and a 500 Hz beep was also presented 200 ms to warn participants that the trial was about to start. The reflection, rotation, or random pattern then appeared on the screen. Following image presentation, participants were prompted to type their duration estimate in a dialogue box. No performance feedback was given.

### Analysis

Mean duration estimates were obtained in each condition for each participant. These data points were then analysed with a 3 × 5-way repeated measures analysis of variance (ANOVA; 3 pattern type [reflection, rotation, and random] × 5 duration [500, 750, 1000, 1250, and 1500 ms]). The Greenhouse-Geisser correction factor was applied when the assumption of sphericity was violated. We report partial η^2^ values following significant effects.

### Results

**Figure 3.**
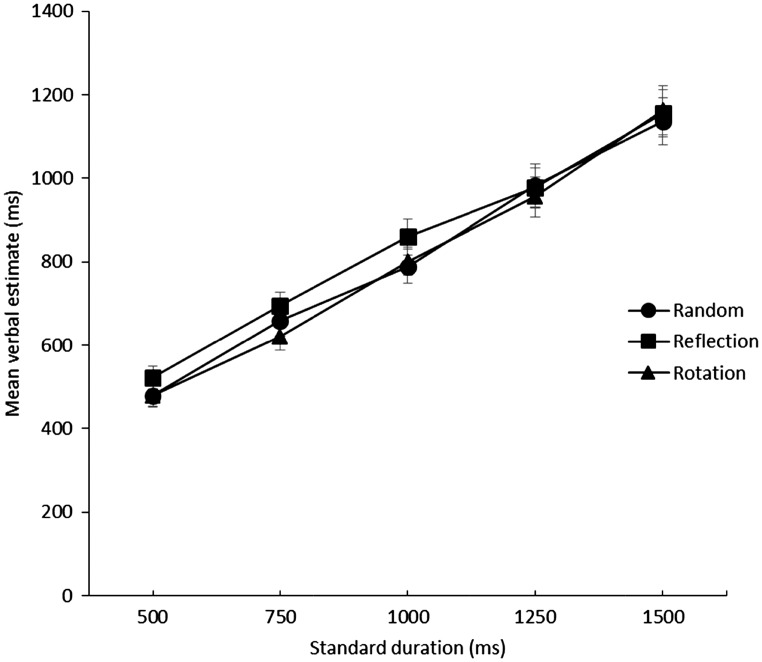
Mean verbal estimates (ms) plotted against the standard duration (ms). Error bars show standard error.

Figure 3 shows mean verbal estimates plotted against presentation duration for the three conditions (reflection, rotation, and random). Longer estimates were given for reflection images, than rotation or random images, but only at shorter durations. There was no difference in perceived duration at longer durations.

A 3 × 5 repeated measures ANOVA revealed significant main effects of duration, *F*(4, 84) = 157.53, *p* < .001, $\eta_{\text{p}}^{2}$^ ^= .88, and pattern type, *F*(2, 42) = 17.24, *p* < .001, $\eta_{\text{p}}^{2}$^ ^= .45. The interaction between pattern type and duration was also significant, *F*(8, 168) = 2.10, *p* < .05, $\eta_{\text{p}}^{2}$^ ^= .09. Post hoc tests (Bonferroni corrected) confirmed that significantly longer estimates were given for reflection than random or rotation (*p* < .001). There was no significant difference between estimates for random and rotation images (*p* = .99).

Further post hoc tests (Bonferroni corrected) showed significantly longer estimates for reflection than random for the 500 ms, and 1000 ms presentations (*p* < .05), there was also a trend for the 750 ms (*p* = .055); however, there was no significant difference in estimates for the 1250 and 1500 ms presentations (*p* > .05). There were also significant differences between reflection and rotation for the 500, 750, and 1000 ms presentations (*p < .*05), but no significant difference for the 1000 and 1500 ms presentations (*p* > .05). Furthermore, there was no significant difference in estimations given for random and rotation at any duration (*p* > .05). Reflectional symmetry lengthened verbal estimates but only when the stimuli were displayed for less than 1 second.

To further analyse the difference across conditions, individual linear regressions were conducted on the mean verbal estimates produced by each participant for each condition. This allowed us to examine the slope and the intercept of the functions. As shown in Figure 3, the slope and intercept of the rotation and random conditions are comparable. Reflection has a higher intercept but a shallower slope. A repeated measures ANOVA found a significant difference in the intercepts of the random (*M* = 152.82, *SD* = 167.75), reflection (*M* = 220.31, *SD* = 171.58), and rotation (*M* = 121.36, *SD* = 163.53), *F*(2, 42) = 10.74, *p* < .001, $\eta_{\text{p}}^{2}$^ ^= .34. Bonferroni corrected post hoc tests showed that intercepts were significantly greater in the reflection condition than the random or rotation conditions (*p* < .01), there was no difference between random and rotation (*p* = .53). The same analysis conducted on the slope of the gradients showed no significant difference in the slope for the random (*M* = .65, *SD* = .23), reflection (*M* = .62, *SD* = .21), and rotation (*M* = .68, *SD* = .24) images, *F*(2, 42) = 2.96, *p* = .06, $\eta_{\text{p}}^{2}$^ ^= .12.

### Discussion

Experiment 1 demonstrated that reflectional symmetry was perceived as lasting for longer than rotational symmetry or random. This therefore replicates the findings of Palumbo et al. (2015). It also confirms that temporal distortions to the perceived duration of visual stimuli can occur in the absence of explicit semantic content and changes in the size, luminance, and colour of the stimuli. Interestingly, reflectional symmetry only lengthened perceived duration when the stimuli were presented for less than 1 second. This is consistent with the sub-second arousal effects found by Gil and Droit-Volet (2012).

The absence of temporal distortion for rotational symmetry is noteworthy. Rotation is equally regular in terms of rigid transformation (Mach, 1886/1959). However, we presume that rotation was less perceptually obvious than reflection for our participants. Ideally, we would have tested this assumption by running a secondary regularity discrimination experiment using the same stimuli and same participants. However, this was probably not necessary. We are confident that the perceptual advantage for reflection over rotation is near-universal for human observers. The salience of reflection is immediately apparent when looking at example patterns like those in Figure 2 (Julesz, 1971; Mach, 1886/1959). This difference between reflection and rotation has been confirmed in numerous psychophysical studies (for early e.g., see Palmer & Hemenway, 1978; Royer, 1981 and other reviewed in Wagemans, 1995). Furthermore, it has been found that reflection produces a larger response than rotation in the extrastriate symmetry sensitive network (Makin, Rampone, Pecchinenda, & Bertamini, 2013; Makin, Wilton, Pecchinenda, & Bertamini, 2012). Formal models of “perceptual goodness” also assign lower scores to rotation than reflection (van der Helm & Leeuwenberg, 1996), and these models have been empirically validated (e.g., Nucci & Wagemans, 2007). It may thus be that rotation does not have the same effect on subjective duration as reflection because it was less perceptually obvious.

To explore whether the results of Experiment 1 were an artefact of the verbal estimation procedure, an Experiment 2 was conducted employing a paired-comparison methodology. One potentially problematic feature of verbal estimation tasks like the one used in Experiment 1 is that participants tend to *quantize* their responses—that is, they are far more likely to enter an estimate which ends in “00.” This behaviour has systematic consequences on the variability of verbal estimates (which may otherwise remain a fixed proportion of the mean, see Wearden, 2015). Although quantization may not distort mean duration estimates (Wearden, 2015), it is prudent to replicate the results Experiment 1 with a different procedure.

## Experiment 2

Experiment 2 tested whether the effect of reflection symmetry on perceived duration could be replicated using a different paradigm. Of particular interest was whether reflectional symmetry would be perceived as lasting for longer than nonsymmetrical stimuli when presented for short (<1 second) but not long (>1 second) durations. A modified version of the paired-comparison task used in Wearden and Ferrara (1993) was used. Participants were presented with two images (one with a symmetrical configuration and the other with a random configuration), and they indicated which one lasted for longer, the first or the second. In some trials, the first and second images were of differing durations (difference trials: 1 < 2, 2 > 1); in other trials, both images were presented for the same amount of time (same trials: 1 = 2). The responses on same trials were most interesting, as this show differences in the perceived duration of reflection and random images. Based on the findings of Experiment 1, we anticipated that there would be a greater proportion of reflection than random stimuli chosen when the stimuli were presented for short (<1 second) durations.

### Method

#### Participants, stimulus, and apparatus

Twenty-four participants (M age = 18.96 years, 15 females, *SD* = 1.30) took part in Experiment 2. All participants had normal or corrected-to-normal vision. Participants received £5 for participation. The stimuli and apparatus were the same as in Experiment 1, however only reflection and random images were used.

### Procedure

Participants were seated approximately 60 cm from the computer screen. They were instructed that they would be presented with pairs of images, and that their task was to indicate which image was presented for longer, the first or the second image. Participants first completed a practice session consisting of 24 trials. Participants then completed a further six blocks of 48 experimental trials.

At the start of each trial, a fixation cross appeared in the centre of a grey screen for 1000 ms. Following this, Image 1 was presented, followed by a delay of 500 ms, then Image 2 was presented. On 50% of trials, Image 1 depicted reflectional symmetry, and on the other 50%, it was a random pattern. Participants were then instructed to indicate which image lasted for longer (by pressing 1 for Image 1 and 2 for Image 2). No performance feedback was given. All trials were presented in a random order.

There were 144 short trials and 144 long trials. On 50% of the trials, the images in each pair differed in duration (different trials). One image in each pair was labelled as the standard and the other the comparison. In short trials, the standard was presented for 400, 500, or 600 ms. In long trials, the standard was presented for 1400, 1500, or 1600 ms. The duration of the comparison was calculated by multiplying the standard duration by .70, .80, .90, 1.10, 1.20, and 1.30. Whether the standard or the comparison was presented first was counterbalanced across the other independent variables in the experiment. On 50% of the trials, both images were the same duration (same trials). The interesting metric is the proportion of reflection and random patterns judged to have been presented for longer when they were presented for the same duration. Given the results of Experiment 1, we hypothesised that “p longer” would be significantly greater for reflection images than random images but only in the short duration range.

### Results

**Figure 4.**
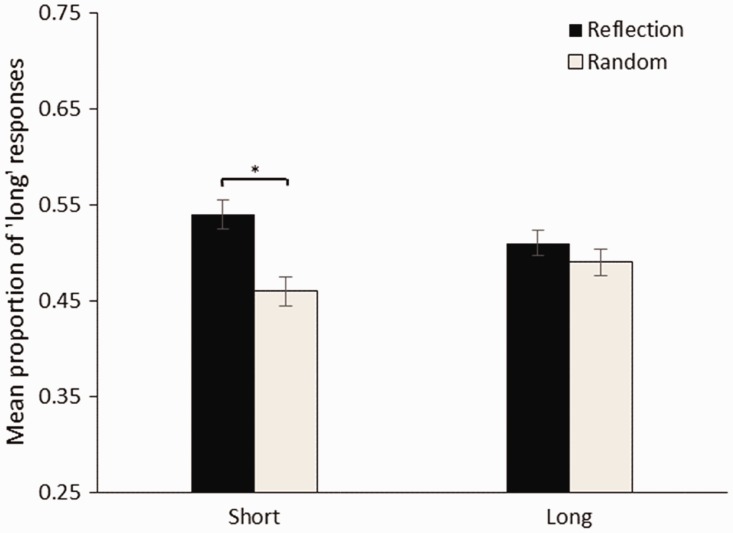
Mean proportion of long responses for reflection and random in the short (400–600 ms) and long (1400–1600 ms) conditions. Error bars show standard error. *p < .05.

First, data from “same” trials were analysed. Figure 4 shows the mean proportion of times that reflection and random images were judged to be longer for the short and long trials. For the short duration range, a binomial test showed that the proportion “reflection long” responses (0.53) was significantly greater than the expected proportion (0.50), *p* = .004. For the long duration range, a binomial test showed that the proportion “reflection long” responses (0.51) was not significantly different to the expected proportion (0.50), *p* = .24. However, we note that there was not a significant difference in the proportion of long responses for reflection between the long and short conditions, *t*(23) = 1.14, *p = *.17.

**Figure 5.**
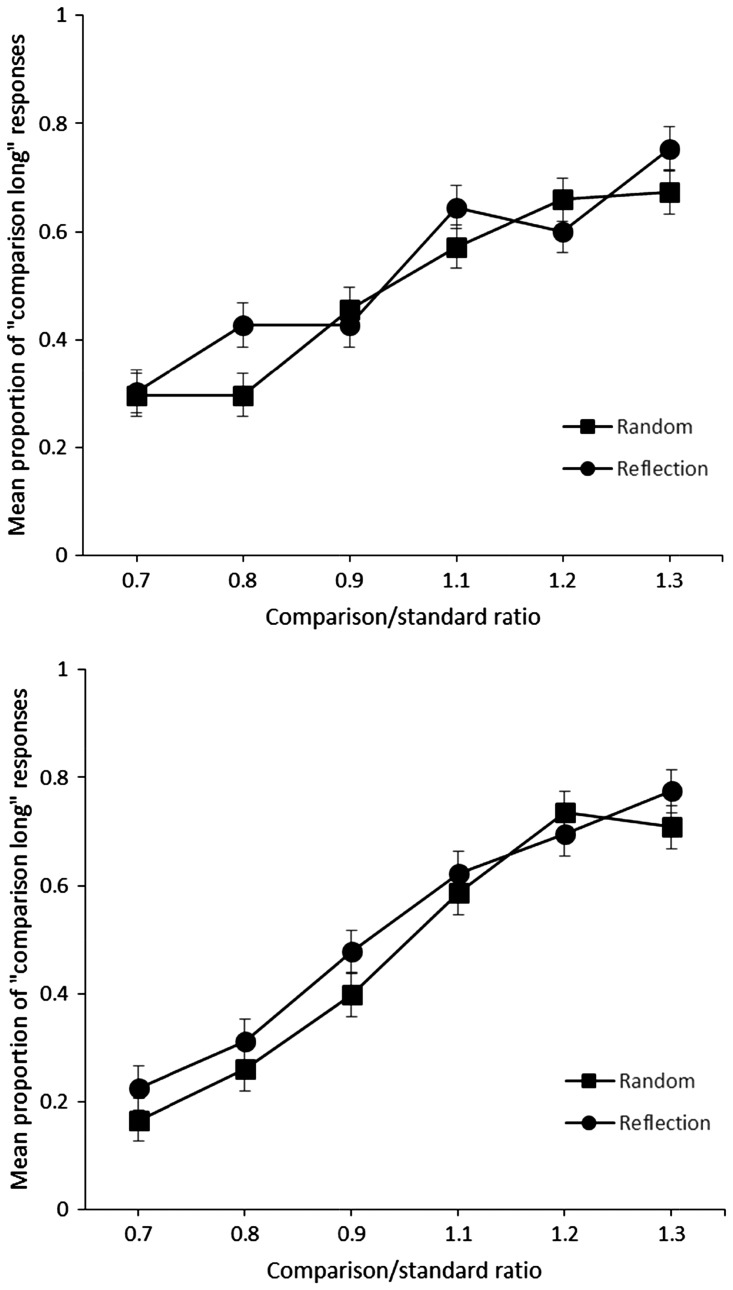
Mean proportion of “comparison long” responses plotted against the comparison or standard. Upper panel shows data from the short duration range and lower panel shows data from the long duration range.

Figure 5 shows the proportion of times that the comparison was selected as longer for random and reflection images for different trials. The upper panel shows data from the short duration range, and the lower panel shows data from the long duration range. Data from each duration range were analysed separately. For the short duration range, a repeated measures ANOVA with within-subject factors of image type (random vs. reflection) and standard or comparison ratio (0.7, 0.8, 0.9, 1.1, 1.2, 1.3) showed a significant main effect of comparison or standard ratio, *F*(5, 110) = 33.08, *p* < .001, $\eta_{\text{p}}^{2}$^ ^= .60. There was no significant effect of image type, *F*(1, 22) = 1.08, *p* = .31, $\eta_{\text{p}}^{2}$^ ^= .05, the interaction between image type and comparison standard ratio was also not significant, *F*(5, 110) = 2.08, *p* = .07, $\eta_{\text{p}}^{2}$^ ^= .08. The same analysis conducted on the long duration range similarly showed a significant main effect of comparison or standard ratio, *F*(5, 110) = 51.06, *p* < .001, $\eta_{\text{p}}^{2}$^ ^= .69. There was no significant effect of image type, *F*(1, 22) = 3.83, *p* = .07, $\eta_{\text{p}}^{2}$^ ^= .14, the interaction between image type and comparison standard ratio was also not significant, *F*(5, 110) = .85, *p* = .51, $\eta_{\text{p}}^{2}$^ ^= .04.

### Discussion

Experiment 2 confirmed that reflectional symmetry was perceived as lasting for longer than random, replicating the lengthening effect observed in Palumbo et al. (2015) and Experiment 1. For “same trials” at short durations (<1 second), reflection was judged to last for longer than random. When the images were displayed for long durations (>1 second), there was no difference in the perceived durations. This also confirms the findings of Experiment 1: symmetry affects duration judgements at short (<1 second) but not long durations (>1 second), which is consistent with the work of Gil and Droit-Volet (2012). These effects should be treated with some caution because the difference between long and short conditions was not itself statistically significant (see Nieuwenhuis, Forstmann, & Wagenmakers, 2011, for discussion of the dangers of over interpreting this scenario). When the images were of different durations, however, there was no effect of image type on responding.

## General Discussion

Experiments 1 and 2 demonstrated that the perceived duration of a visual stimulus can be distorted by the presence of symmetry. In Experiment 1, images depicting reflectional symmetry were estimated as lasting for longer than rotation and random patterns. In Experiment 2, reflectional symmetry was perceived as lasting for longer than random patterns when the actual presentation duration was identical. In both experiments, this lengthening effect is only occurred when the stimuli were presented for less than 1 second. The effect is not therefore an artefact of either experimental paradigm. Symmetry, therefore, lengthens the subjective presentation duration of short, but not long, images. Critically, these distortions existed in the absence of changes in the stimulus luminance, size, and colour, and in the absence of explicit semantic content within the image being judged. These experiments therefore demonstrate that affective modulation of timing is not dependent on changes in lower level image properties, or explicit semantic content.

The subjective lengthening of the duration of reflectional symmetry is consistent with other reports of longer perceived duration for low-arousal positively valenced images (Angrilli et al., 1997; Droit-Volet et al., 2004; Gil & Droit-Volet, 2012; Smith et al., 2011) . Like other forms of low-arousal positively valenced stimuli, reflection may have lengthened subjective estimates of duration because it increased arousal. According to SET, the rate that the pacemaker emits output is arousal sensitive, so arousing stimuli are judged as lasting longer. Reflectional symmetry is aesthetically pleasing and is preferred by humans (Cardenas & Harris, 2006; Eisenman, 1967; Eysenk, 1941; Frith & Nias, 1974; Jacobsen & Höfel, 2002) and animals (Wignall et al., 2006) . In humans, this preference has been demonstrated explicitly and implicitly (Makin et al., 2012). The increased arousal elicited by reflectional symmetry, as opposed to rotational symmetry or random, may have led to an increase in pacemaker output rate and a longer perceived duration.

The effect of arousal on pacemaker rate is typically thought of as multiplicative (i.e., the increased rate has a larger effect at longer durations) which manifest as condition-based differences in the slope of the estimation gradient. In the current study, however, symmetry only consistently lengthened duration estimates when the stimuli were presented for less than 1 second, no lengthening effect was observed for stimuli presented for longer than 1 second. Consequently, the difference in the slope of gradients was not significant. There was however a significant difference in the intercepts of the gradients. This is not the first instance in which intercept differences have been observed in the absence of slope differences when comparing purportedly arousing and neutral stimuli (Jones & Ogden, 2016; Makin, Lawson, Bertamini, & Pickering, 2014; Makin, Poliakoff, et al., 2012). Indeed some studies demonstrating lengthening of the perceived duration of arousing stimuli show no difference in the slope and intercept of the verbal estimation gradients (Gil & Droit-Volet, 2012), whereas others show significant differences in both the slope and the intercepts of the gradients (Ogden et al., 2015). The absence of consistent slope and intercept differences, despite evidence of subjective lengthening, supports Matthews (2011) caution against the use of slope and intercept information alone as indicators of internal clock effects.

The time-limited effects observed in this and other studies using visual stimuli (e.g., Gil & Droit-Volet, 2012) contrast markedly with the effects of increased arousal on the perceived duration of arousing auditory and somatosensory stimuli (Fayolle et al., 2015; Ogden et al., 2015) where greater effects are clearly observable at durations beyond 1 second. This cross-modal comparison may suggest that arousal effects for visual stimuli decay more quickly than for stimuli of other modalities. However, we caution that the term “arousal” has several meanings in neuroscience. It is unlikely that mere presentation of visual reflectional symmetry produced adrenaline release and activation of sympathetic nervous system. Reflection might produce cortical arousal, perhaps via reduction in alpha oscillations (e.g., Klimesch, Sauseng, & Hanslmayr, 2007). Of course, we did not record any form of arousal independently here, so the claim that reflection increased subjective duration via arousal is circular. We also note that different cognitive mechanisms may be required for timing sub- and supra-second stimuli. There is a debate about whether there is a central, supramodal clock in the brain, as SET implies, or whether multiple timing systems are recruited in difference modalities and dimensions (Johnston et al., 2006, Van Rijn & Taatgen, 2008). Coull, Cheng, and Meck (2011) reviewed recent literature and concluded that the results are consistent with a supramodal clock in the dorsal striatum of the basal ganglia and presupplementary motor area, but that distributed timing mechanisms may dominate in the sub-second range. This all remains controversial. However, it is interesting that these results tentatively support a dissociation between effects sub- and supra-second intervals.

The fact that subjective lengthening was only found for reflection, and not rotation is interesting. We speculate that this is because reflectional symmetry is biologically relevant, and more likely to produce an emotional response. However, we also note that reflectional symmetry is more obvious to human observers than rotation, even when the number of rigid transformations is identical (Mach, 1886/1959; Royer, 1981). It could be that the magnitude of temporal distortion is proportional to the salience of the regularity. This account predicts that any manipulation that varies perceptual goodness of the patterns should alter perceived duration in a systematic way. For example, reflection with more axes should be perceived as lasting longer (cf. van der Helm & Leeuwenberg, 1996). This could be tested in future work. The results of Experiment 1 suggest that reflection was responsible for lengthening subjective duration (rather than random shortening subjective duration). With the rotation condition, it would be impossible to discriminate between these accounts. Further work will be required to isolate the specific property of reflection responsible for this effect.

## Conclusions

Images displaying reflectional symmetry are perceived as lasting for longer than images displaying rotational symmetry or random configurations. This lengthening effect only consistently occurs however for images presented for less than 1 second. The presence of symmetry has little effect on images presented for longer than 1 second. This suggests that arousal effects of visual stimuli may decay more quickly than for other modalities. Distortions to the perceived duration of an image can occur in the absence of explicit semantic content and in the absence of physical changes in image, luminance, and size.
